# Smartphone-based multispectral autofluorescence analysis of bacteria mixtures of staphylococci using convolutional neural network

**DOI:** 10.1186/s13036-025-00616-7

**Published:** 2026-01-23

**Authors:** Jocelyn Reynolds, Katelyn Sosnowski, Christine Carlson, Thomas D. McGuire, Will Roman, Jeong-Yeol Yoon

**Affiliations:** Department of Biomedical Engineering, The University of Arizona, Tucson, AZ 85721 USA

**Keywords:** Smartphone microscope, Skin swab, *Staphylococcus aureus*, Machine learning, CNN

## Abstract

Despite the numerous merits of bioreceptor-based biosensors for detecting bacteria, they often fail to identify complex bacterial mixtures, such as those encountered in microbiome analysis, especially when the bacterial mixtures belong to the same genus. Additionally, their procedure involves multiple steps of reagent rinsing and/or staining. This work demonstrates the proof-of-concept for smartphone-based multispectral autofluorescence analysis of bacterial mixtures belonging to the same genus: *Staphylococcus epidermidis*, *Staphylococcus haemolyticus*, *Staphylococcus capitis*, and *Staphylococcus aureus*, that does not require bioreceptors or staining processes. These staphylococci mixture models represent the skin infection model, where the goal is to identify the presence of pathogenic *S. aureus* (coagulase-positive) from other *Staphylococcus* spp. (coagulase-negative staphylococci or CoNS). We designed and tested a portable, inexpensive smartphone-based imaging platform using a smartphone microscope attachment with a 3D-printed housing, LEDs for sample excitation, and low-cost color films for filtering fluorescent emission. For each data point, the excitation light sources and emission filters were alternated by sliding the color films into place and pressing the LED buttons to acquire nine unique autofluorescence images. A convolutional neural network (CNN) model was developed for various bacterial mixtures of CoNS and CoNS + *S. aureus*. It demonstrated excellent performance in detecting *S. aureus* presence, with a sensitivity of 91%. However, a small number of false positives compromised the overall accuracy to 84%. Experiments were further replicated with the CoNS- and *S. aureus*-spiked human skin swab samples. While the sensitivity (for identifying *S. aureus*) remained satisfactory at 81%, the overall accuracy was compromised to 68% due to the substantial number of false positives. This could be attributed to the inherent limitations of the currently available smartphone-based microscope attachment, which makes focusing challenging, especially with skin swab samples. Overall, this work provides a proof-of-concept for identifying bacterial mixtures belonging to the same genus without using bioreceptors or laboratory equipment, toward low-cost, rapid assessment of complex bacterial mixture samples.

**Supplementary Information:**

The online version contains supplementary material available at 10.1186/s13036-025-00616-7.

## Introduction

Numerous biosensors have been developed to detect and quantify bacteria across a wide range of samples, including environmental (water and soil), agricultural (animals and plants), and human (saliva, urine, feces, blood, etc.). These biosensors typically utilize specific bioreceptors, such as antibodies, aptamers, nanozymes, molecularly imprinted polymers, and nucleic acid sequences, among others [1, 2]. The specific binding of target bacterial species to these bioreceptors is identified and quantified using various transducers, including electrochemical or optical transducers [1, 2].

These biosensors can also be used to detect and classify complex mixtures of bacteria, such as various types of microbiota. Recently, there has been a growing interest in microbiome analysis, including environmental (soil), agricultural (animals and plants), and human (skin, gut, oral cavity, lung, etc.) applications [3]. Previously, characterization of these microbiomes has typically been achieved by next-generation sequencing (NGS) [4], as well as metabarcoding and metagenomics [5]. While these laboratory-based analyses have been proven exceptionally powerful in accurately characterizing the makeup of complex microbiota, they still suffer from limitations associated with the use of expensive instruments, the need for highly trained personnel, and lengthy assay times [6, 7]. Bioreceptor-based biosensors can potentially eliminate such complications related to instrumentation, training, and assay time.

However, bioreceptors often fail to identify and classify the complex microbiota. For example, over 200 genera and 18 phyla have been reported in the skin microbiome [8, 9]. In addition, most bioreceptors are known to cross-bind to different subspecies and even to similar species within the same genus, especially within complex bacterial mixtures [4]. In the skin microbiome, a mixture of *Staphylococcus* spp. is commonly found, including *Staphylococcus epidermidis*, *Staphylococcus haemolyticus*, and *Staphylococcus capitis*, which are collectively referred to as coagulase-negative staphylococci (CoNS) [10–14]. The presence of pathogenic species, such as *Staphylococcus aureus* (referred to as coagulase-positive staphylococci or CoPS), triggers a pathogenic response from the host (i.e., atopic dermatitis) [15]. Therefore, bioreceptor-based biosensors may not yield results as satisfactory as laboratory-based sequencing methods. Besides, biosensors still require bioreceptors, which can be costly, especially when a large number of bioreceptors are needed. Their detection processes can involve multiple reaction steps and a large number of wells or channels, augmenting the complexity of such systems.

There is a need for a technology that can analyze bacterial mixture composition in a non-invasive, cost-effective, and rapid manner, enabling better understanding of bacterial mixtures and potentially serving as a tool for monitoring their pathogenicity. We hypothesize that we can enhance bacterial mixture identification using multispectral autofluorescence imaging, without relying on bioreceptors or staining processes, and then apply machine learning classification. Such a method can be relatively quick and inexpensive without requiring complicated laboratory procedures (such as genomic sequencing) or lengthy assay times. We aim to utilize bacterial autofluorescence, i.e., without using bioreceptor-fluorescent-dye-based staining. For decades, autofluorescence has been used as a simple diagnostic technique using an ultraviolet device known as a Wood’s Lamp [16]. This device can distinguish between some cancerous and infectious skin conditions, but it is not optimized for characterizing subtle differences in the complex microbiome.

This work aims to identify and classify bacterial mixtures composed of bacteria within the same genus (*Staphylococcus* spp.) using a low-cost, portable device as an alternative to sequence-based techniques. Our group has previously captured fluorescence or autofluorescence signals from bacteria, oil spills, and fluorescent microparticles using custom-built, inexpensive technology that utilizes light-emitting diodes (LEDs) and simple color films in place of lasers and high-end glass filters [17–19]. Using smartphones for data collection enables point-of-care analysis by eliminating the need to purchase large laboratory equipment. This work optimized a similar platform for analyzing bacterial mixtures, which are modeled with the skin microbiome. It utilized a series of color films and stacks of autofluorescence images and analyzed them using machine learning. A significant training database of images was compiled from the bacterial mixtures. The autofluorescence signal was expected to be relatively weak, especially considering the low bacterial concentrations, which matched those found in the skin microbiota. Nonetheless, this approach sought to avoid a specific staining procedure, aiming to create a portable, rapid analytical method. Staining would add reagents, time, and expense. The most challenging issue is identifying *S. aureus* among a myriad of other bacterial species, which has not been demonstrated in the previous works [17–21]. Such a challenge is attributed to the non-specific nature of autofluorescence, as many biological molecules in microbiota emit autofluorescence. Despite these difficulties, algorithms known as neural networks are particularly well-suited for pattern recognition tasks of this nature [22, 23]. Hence, we aim to demonstrate that a convolutional neural network (CNN) can recognize patterns that identify *S. aureus* (a pathogenic bacterium) in the presence of other *Staphylococcus* spp. mixtures (CoNS), based on nuanced differences in their autofluorescence signals as collected with our custom-built smartphone microscope device.

Next, as an example of a complex bacterial mixture, we non-invasively obtained skin swab samples from the antecubital fossa (inner elbow) region of healthy volunteers. The right-arm samples were subsequently spiked with *S. aureus*, and the left-arm samples with CoNS bacteria. We then imaged the samples using our device to test whether a newly trained algorithm could distinguish between *S. aureus*-spiked and CoNS-spiked skin swab samples. This work represents a significant step toward a portable, rapid methodology for classifying bacterial mixtures belonging to the same genus, which is closely linked to microbiome analysis.

## Materials and methods

### Bacteria culture

Bacterial samples were prepared by culturing bacteria in liquid medium and staining microscope slides for imaging. Tryptic soy agar (TSA) was prepared using 3 g of tryptic soy broth powder (NutriSelect^®^; MilliporeSigma, Burlington, MA, USA) and 2 g of agarose (type I – a low EEO; Sigma-Aldrich, St. Louis, MO, USA) per 100 mL of deionized (DI) water sterilized by autoclave. The bacteria *S. aureus*,* S. epidermidis*, *S. haemolyticus*, and *S. capitis* were separately inoculated onto TSA plates using KWIK-STIK™ devices (ATCC^®^ 12600TM, 12228TM, 29970TM, 35661TM, and BAA-1293TM, respectively; Microbiologics, St. Cloud, MN, USA). Sterile plastic inoculation loops (inoculating loops/needles clear; Thermo Fisher Scientific, Waltham, MA, USA; inoculation loops; SARSTEDT, Nümbrecht, Germany) were used to spread the bacteria across the plates after using the KWIK-STIK™ for application. TSA plates were incubated at 37 °C for 24 h (*S. aureus*, *S. epidermidis*, and *S. haemolyticus*) or 48 h (*S. capitis*). A control plate “inoculated” with an empty inoculation loop was incubated alongside the bacterial plates to rule out any contamination. Next, a single colony was isolated from each plate and transferred to separate 10 mL tubes of tryptic soy broth, prepared from the same recipe as TSA, but without the agarose. These were incubated at 37 °C for 24 h alongside a control tube of 10 mL tryptic soy broth “inoculated” with an empty inoculation loop to rule out contamination. Samples were then analyzed with a miniature spectrophotometer and software (USB4000 and OceanView Lite version 1.6.7; Ocean Insight, Orlando, FL, USA) to confirm bacterial concentration using the absorbance at 600 nm according to the McFarland standard [24, 25]. Samples were kept for use only if the control tube showed no bacterial growth. Samples were stored in 1 mL aliquots in dimethyl sulfoxide (DMSO; Mediatech, Manassas, VA, USA) at a ratio of 850 µL culture in 150 µL DMSO and kept in a − 40 °C deep freezer until used for imaging experiments. DMSO served as a cryoprotective agent, preserving bacterial viability during freezer storage [26]. When stored under proper conditions, bacteria can remain viable for one to three years at − 40 °C and up to 10 years at − 80 °C [27]. The cultured bacteria were stored for less than 12 months at − 40 °C to ensure the viability of the bacteria.

### Device construction

**Fig. 1:**
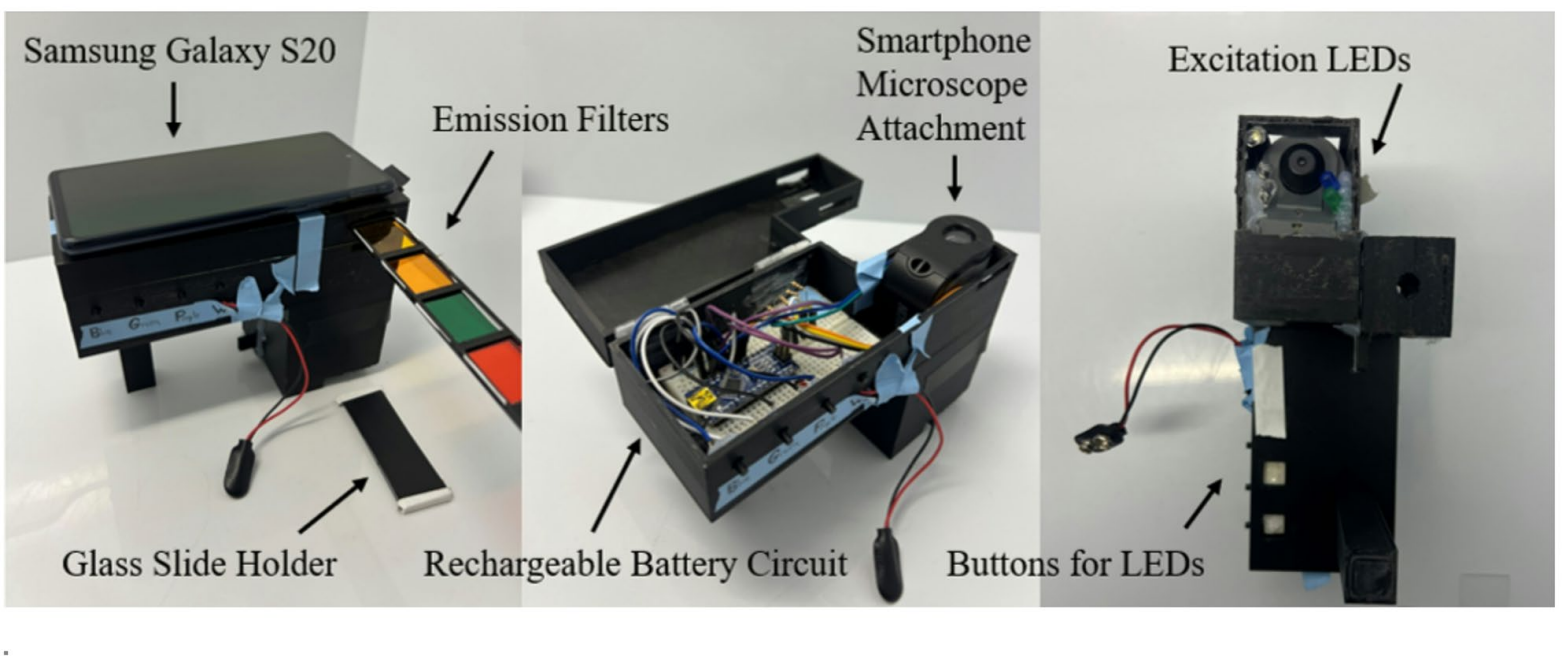
Custom-built device for autofluorescence imaging of skin bacterial samples

Figure 1 shows the completed device. The housing was designed in SolidWorks (SolidWorks 2019 SP5.0; Dassault Systèmes, Waltham, MA, USA) and 3D-printed using black polylactic acid (PLA) filament (PLA 1.75 mm true black; Hatchbox 3D, Pomona, CA, USA) on a Creality Ender 3 printer (Shenzhen Creality 3D Technology, Shenzhen, China). Circuitry included 405 nm, 460 nm, and 489 nm LEDs connected in parallel to a 9 V rechargeable battery (EBL Official; Shenzhen, China) via four-prong buttons (7/16 in square), which connected the circuit between each LED and the battery when pressed. A white LED was also connected to the battery through a switch (16 A 125 V_AC_ T105 rocker switch). The UV-visible spectra of the LEDs can be found in Supplementary Figure S1. A smartphone microscope attachment (MicroFlip MP-250; Carson Optical, Ronkonkoma, NY, USA) was positioned in the main body of the housing with LEDs affixed near the objective, approximately 30 mm above the slide, and angled to focus on the area of the glass slide directly beneath the microscope. This microscope has a magnification range of 100x to 250x with a field of view of 1.1 mm to 0.8 mm. Between the top of the microscope and where the smartphone rests, there are two slits and a small window (2 mm x 20 mm) that allows a custom color filter slider to be inserted, controlling the wavelengths of light collected and imaged by the smartphone. Roscolux color film filters (Rosco Laboratories, Stamford, CT, USA) were selected based on their specific emission wavelength to capture the meaningful autofluorescence emitted by the bacteria. Specifically, six unique filters were used: Yellow R10 – Medium Yellow; Yellow R12 – Straw; Light Orange R15 – Deep Straw; Dark Orange R20 – Medium Amber; Green R2004 – VS Green; Red R22 – Deep Amber. Each filter is used with a unique combination of LED and exposure time to collect meaningful data. Absorbance or emission spectra of the bacterial mixtures were not collected since many bacterial species share common endogenous fluorophores. This results in spectral overlap and a flattened emission spectrum, making it difficult to identify individual bacterial species’ autofluorescence signals without using post-processing techniques such as canonical polyadic decomposition [28].

The LEDs were selected for their ability to excite the endogenous bacterial fluorophores such as porphyrins and flavins, which are excited at 405 nm and emit blue, green, and yellow [29]. Specifically, nicotinamide adenine dinucleotide (NADH) emits blue, flavin emits green, and porphyrins emit red autofluorescence [29]. Furthermore, oxidized flavins, such as flavin adenine dinucleotide (FAD) and flavin mononucleotide (FMN), can be excited at wavelengths between 450 and 490 nm [30]. To isolate these emitted signals from the excitation light, emission filters with distinct transmission profiles were used. The filters were selected through trial and error to determine which ones most effectively blocked the excitation light while allowing the emitted fluorescence signal to pass through. The exposure times in Table 1 were determined empirically by capturing a series of test images at varying exposure durations for each excitation wavelength and emission filter combination. The tested exposure times ranged from 1/4, 1/3, 1/2, 1, 2, 4, 8, 10, 15, 20, to 30 s. Then, these preliminary images were analyzed in ImageJ, where they were converted to the HSV color space. The images were visually inspected in the saturation channel, and the optimal exposure time was determined as the one that resulted in full illumination without gaps, as these gaps could confuse the machine learning model. In addition, the minimum exposure time that yielded no gaps in the saturation channel was selected to minimize image overexposure. This process was then repeated for each LED and filter combination, thereby optimizing exposure times for each image. The microscope glass slide with the bacterial sample is placed on a slide holder and positioned beneath the objective lens so that the droplet is within view. The microscope slide holder is constructed by stacking three glass microscope slides (Fisherbrand Plain Microscope Slides Precleaned; Thermo Fisher Scientific) and painting the glass slide with black paint (Musou Black Water-based Acrylic Paint; Koyo Orient Japan, Saitama, Japan). The slides in the slide holder were coated with Musou Black paint, which absorbs 99.4% of visible light. It prevents light from the slide from scattering or reflecting off the slides beneath the sample, thus ensuring that the smartphone captures only the light produced by the bacteria’s autofluorescence. The Samsung Galaxy S20 FE (SM-G781U1) smartphone rested on top of the device housing just above a window where the film slide and microscope eyepiece are located. Furthermore, the images taken with this smartphone utilized the main wide camera, which has an aperture of f/1.8 and a focal length of 5.4 mm. Additionally, using the smartphone’s ProCamera application, the ISO was fixed to 640 and the white balance to 4000 K. The camera was operated in manual mode with the smartphone’s autofocus disabled, as automatic algorithms adjust exposure and gain automatically, which can cause over- or under-exposed images [31, 32]. In return, the variation in images caused by automatic focusing can interfere with machine learning; therefore, the camera’s autofocusing feature was not utilized. The circuitry, including the rechargeable battery, was easily accessible by lifting the 3D-printed lid of the device, which was attached to the rest of the housing using two plastic hinges (opened dimensions: 33.3 mm x 28.6 mm) attached with superglue (Insta-Cure™ Cyanoacrylate; Bob Smith Industries, Atascadero, CA, USA).

**Table 1:** Device settings for collecting nine autofluorescent images from a single sample aliquot

Image	Excitation LED (nm)	Emission filter	Transmitted wavelengths from emission filter (nm)	Exposure time (s)
***Bacterial mixtures***				
1	405	Yellow (R10 Medium Yellow)	< 390, > 480	2
2	405	Yellow (R12 Straw)	< 400, > 480	2
3	405	Light orange (R15 Deep Straw)	> 500	2
4	460	Light orange (R15 Deep Straw)	> 500	1
5	460	Dark orange (R20 Medium Amber)	< 440, > 500	1
6	460	Green (R2004 Storaro Green)	480–600, > 700	1
7	405	Green (R2004 Storaro Green)	480–600, > 700	4
8	460	Red (R22 Deep Amber)	> 560	4
9	489	Red (R22 Deep Amber)	> 560	4
***Skin swab samples***				
1	405	Yellow (R10 Medium Yellow)	< 390, > 480	2
2	405	Yellow (R12 Straw)	< 400, > 480	2
3	405	Light orange (R15 Deep Straw)	> 500	10
4	460	Light orange (R15 Deep Straw)	> 500	20
5	460	Dark orange (R20 Medium Amber)	< 440, > 500	8
6	460	Red (R22 Deep Amber)	> 560	30
7	489	Red (R22 Deep Amber)	> 560	8
8	460	Green (R2004 Storaro Green)	480–600, > 700	2
9	405	Green (R2004 Storaro Green)	480–600, > 700	2

### Bacterial mixtures

**Fig. 2:**
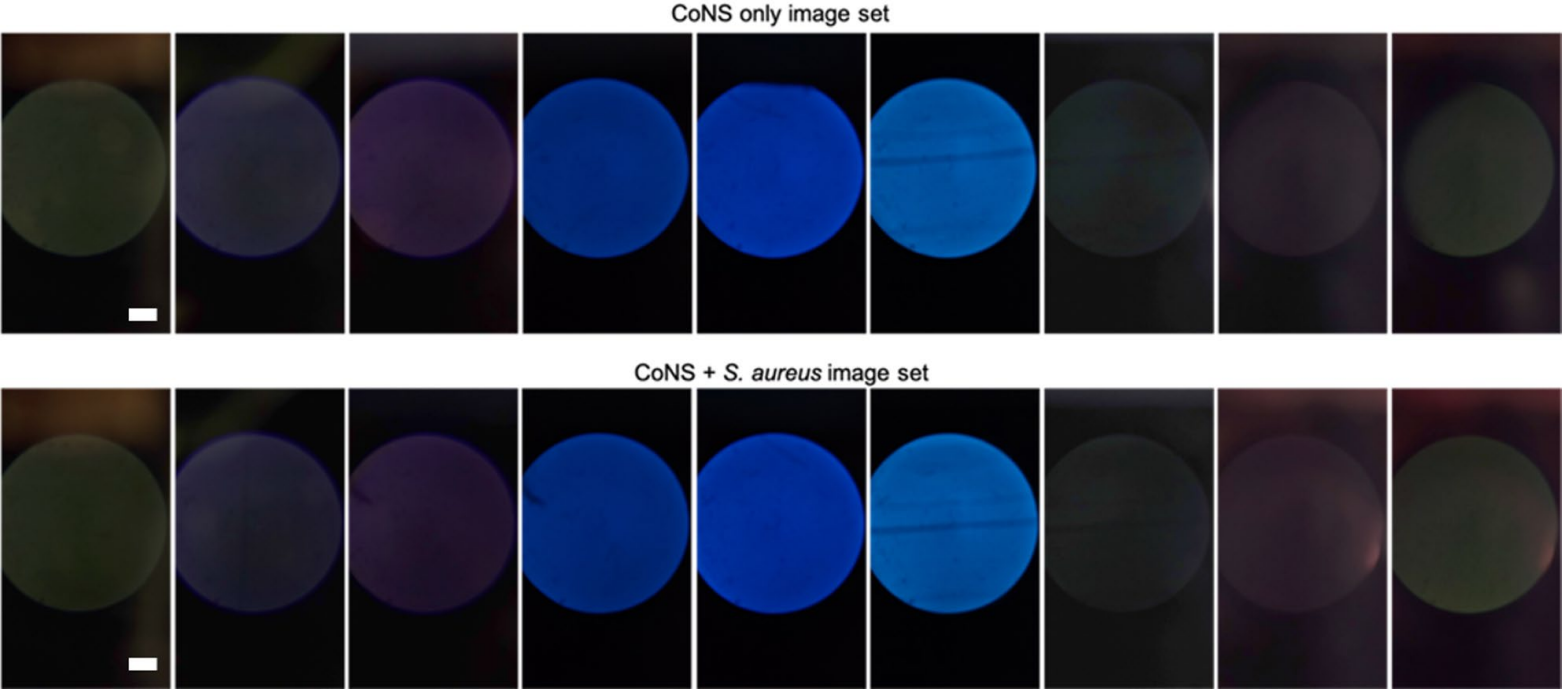
Example images taken with the device. Nine images were taken from a single aliquot of a bacterial mixture, varying LED and filter combinations. From left to right, images 1 through 9, as labeled in Table 1. Visually, CoNS-only and CoNS + *S. aureus* images do not appear much different. Scale bar = 200 μm. Note: Images 1–3 and 7–9 in each set were adjusted for brightness and contrast for visualization purposes in this figure

Frozen samples were thawed to room temperature and vortexed. To preserve the viability, once the bacteria were thawed, DMSO was removed, and the bacterial pellets were resuspended in DI water. All CoNS species (*S. epidermidis*, *S. haemolyticus*, and *S. capitis*) were combined in equal parts into one tube, while *S. aureus* was aliquoted into a separate tube. *S. aureus* and CoNS bacteria were centrifuged at 14,000 x *g* for 1 min to obtain a pellet. Next, the tubes were re-diluted in DI water by removing the liquid portion (without the pellet), adjusting the pipette to remove air bubbles, ejecting the liquid portion, pulling the adjusted amount of DI water with a new pipette tip, and pipette-mixing this volume of water into the tube. In this way, the concentration of bacteria was preserved. Next, DI-diluted samples were mixed to the following ratios: 0% *S. aureus* and 100% CoNS (= CoNS); 50% *S. aureus* and 50% CoNS (= CoPS); and 70% *S. aureus* and 30% CoNS (= CoPS). Both 50:50 and 70:30 mixtures of *S. aureus* and CoNS were used as CoPS samples. All samples were further diluted in DI water to an overall bacterial concentration of 2.2 × 10^7^ bacteria/mL (similar to that of a skin swab sample, i.e., 10^4^ bacteria/cm^2^ [33]. This fixed overall concentration ensured that the autofluorescence signal difference resulted from variations in bacterial composition rather than from overall bacterial concentration. After preparing the bacterial mixtures, 10 µL aliquots of each sample were added to microscope glass slides (Fisherbrand Plain Microscope Slides Precleaned; Thermo Fisher Scientific), which were cleaned using 70% ethanol and a wipe (KimWipes; Kimberly-Clark Professional, Roswell, GA, USA). Three aliquots of the same sample type were placed on the same glass slide. Slides were allowed to dry for at least 1 h before imaging, or, to speed the process, put on a hot plate (MS-H-Pro+; SCILOGEX, Rocky Hill, CT, USA) at 80 °C for a few minutes until dry. A black permanent marker was used to demark the edges of each aliquot for ease of imaging. Then, the smartphone microscope attachment was aligned with the first aliquot using the white LED. Subsequently, a total of nine images were obtained from a single aliquot, as shown in Table 1. LEDs were toggled with buttons, and the filters were changed using the 3D-printed filter slider. This was repeated for each aliquot. Example images are shown in Fig. 2, with magnified versions presented in Supplementary Figure S2. The device’s battery was recharged and replaced daily before each set of experiments, as the device was used.

### Skin swab samples

Human skin swabs were obtained and spiked with CoNS or *S. aureus* as representative examples of clinically relevant *Staphylococci* mixtures. Images were acquired from these samples, and a separate machine learning database and algorithm were developed, as these samples are inherently different from bacterial mixtures. The University of Arizona’s Institutional Review Board (IRB) approved the protocol, designated as STUDY00001560. One swab was collected from each arm using separate sterile nylon flocked swabs, which were stored in liquid Amies medium after the procedure (ESwab™ 480CFA; COPAN Diagnostics, Murrieta, CA, USA). The tube containing the liquid Amies medium and swab was vortexed for 5 s to elute the sample contents into the medium. The swab was then removed, and the tube was placed in the refrigerator for later experiments. Swabs were not pre-wetted with any solution, i.e., a dry swab technique was used. Many skin microbiome studies employ a wet swab technique, but it has been shown that dry swabs can also be effective and may even yield a higher diversity of bacteria [34].

Samples were assumed to contain 2.2 × 10^6^ bacteria/mL, as a skin swab is estimated to acquire ~ 10^4^ bacteria/cm^2^ [33], and the researchers determined that 10 µL covers approximately the area of a standard (2.2 cm^2^) coverslip. Hence, the stock bacterial solutions were diluted to 2.2 × 10^6^ bacteria/mL for spiking into skin swab samples. Again, the concentrations of the bacterial stock solutions were all diluted to the same concentration to ensure that any differences in autofluorescence signals resulted from changes in bacterial composition rather than from the total bacterial amount in the sample.

Skin swab samples were centrifuged and resuspended in DI water, and combined with bacteria samples that were also centrifuged and resuspended in DI water at a 1:1 ratio. Right-arm samples were spiked with *S. aureus*, while left-arm samples were spiked with CoNS. Since skin swab samples are expected to naturally contain CoNS, *S. aureus* was not mixed with CoNS, unlike in the bacterial mixture experiments. However, although the skin swab samples are assumed to contain commensal bacteria, the *S. aureus*-negative samples were still spiked with CoNS to ensure consistent concentrations between the two samples. It prevents biased machine learning due to differences in bacterial concentrations between *S. aureus-*negative and positive samples. Specifically, the frozen bacteria stock solutions resuspended in DI water were used to create CoNS and *S. aureus* mixtures by combining 1989.4 µL DI water with 11.6 µL bacteria. For the CoNS solution, 3.9 µL of each bacterium, *S. epidermidis*, *S. haemolyticus*, and *S. capitis*, was mixed together. For the *S. aureus* solution, 11.6 µL *of S. aureus* was added to the DI water. Once these two bacterial solutions were prepared, 250 µL of the solution was added to the skin swabs, with *S. aureus* spiked into the left arm sample and CoNS into the right arm sample. Since the skin swab samples were deliberately spiked with either CoNS or *S. aureus*, other microbiological methods were not used to validate the bacterial composition of the skin swab samples. This resulted in an estimated concentration of 2.2 × 10^6^ bacteria/mL in the samples. Next, microscope slides were prepared and imaged as described in the previous section about simulated samples. Again, nine images were taken for each sample on the microscope slides; however, the LED wavelengths were slightly altered this time, which is summarized at the bottom of Table 1. For the human skin swab samples, 30 microscope glass slides were imaged for both *S. aureus* and CoNS samples, with each slide containing three replicates.

### Image pre-processing

The hue-saturation-value/brightness (HSV or HSB) color space was used instead of the red-green-blue (RGB) of the original images because HSV allows for the extraction of meaningful color information in autofluorescence settings [35, 36], as the coloration, richness/opacity, and lightness/darkness of each pixel can be separated from each other. The color films used for autofluorescence imaging restrict the hue and, to some degree, the brightness of the resulting images. Hence, the saturation channel for all nine excitation-emission combinations was used for both algorithms described below. All raw RGB images were converted to HSV space, the saturation channel was isolated, and image patches were created as training data. There were nine excitation-emission combinations (according to Table 1) for each image patch, and they were stacked together as shown in Supplementary Figure S3. Images from the bacterial mixtures (the training dataset) were augmented two-fold by adding 90-degree-rotated images. On the other hand, images from the human swab samples were augmented after splitting the data into training and test sets. The data was augmented fourfold by rotating 90, 180, and 270 degrees due to the small dataset size. Supplementary Figure S3 provides an overview of the data collection and analysis, including image pre-processing steps.

### Linear discriminant analysis (LDA)

To understand the classification problem, we used a simpler (i.e., non-deep learning) method, linear discriminant analysis (LDA), to classify autofluorescence images as *S. aureus-*negative or positive. This simple method was used to analyze only the autofluorescent intensities within the areas of interest, dismissing the morphological data commonly analyzed with CNN (described in the next section). First, images were uploaded into ImageJ software (version 1.51r; National Institutes of Health, Bethesda, Maryland, USA) and converted to HSV color space. The saturation channel was isolated, and an ImageJ macro was used to create a 1851-pixel-diameter circle (approximately 38 μm) centered on the microscope lens and to calculate the average saturation within that region. This process was repeated for all nine LED and emission filter combinations, yielding a total of nine saturation values per sample. These saturation values served as the input features for the LDA classification. Images that could not be analyzed for average saturation (Supplementary Figure S4) were assigned a value of − 1 instead of an average saturation. This data was saved as a .csv file containing all nine saturation features and loaded using Python (version 3.8.2) within a virtual environment on an account through the University of Arizona’s high-performance computing (HPC) system. Code was written in Python to perform LDA using repeated stratified k-fold cross-validation with 10 splits and three repeats (Supplementary Code S1). Specifically, stratified 10-fold cross-validation was used, which splits the data into 10 folds while preserving class balance in each fold. In each run, one fold was used as the testing set, and the remaining nine folds were used for training, ensuring that the test dataset remained unknown. Essentially, 90% of the data was used for training and 10% for testing in each round. The LDA dataset consisted of 100 samples: 40 *S. aureus*-negative CoNS mixtures (label 0) and 60 *S. aureus*-positive (CoNS + *S. aureus*) mixtures (label 1). Moreover, of the 60 positive mixtures, 30 were at a 70:30 *S. aureus* to CoNS ratio, while the remaining 30 were at a 50:50 *S. aureus* to CoNS ratio. LDA used 10-fold cross-validation; therefore, for each run, 90% of the data was used for training, while 10% was held out as the test dataset. With stratification, each dataset contained 40% negative and 60% positive to match the class ratio of the entire dataset. The training dataset consisted of approximately 36 negative and 54 positive mixtures, while the testing dataset contained approximately four negative and six positive mixtures. Average accuracy was reported, and the code was run 10 times.

### Convolutional neural network (CNN)

For convolutional neural network (CNN) analysis of bacterial mixtures, images were uploaded to the HPC account using Globus (version 3.1.6 and 3.2.1.677; University of Chicago, Chicago, IL, USA, and Argonne National Laboratory, Lemont, IL, USA). Python code was run within a virtual environment on the HPC account (Supplementary Code S2). The HPC system was used to process all the data, due to the large number of images and, subsequently, image patches used to train and test the model. The HPC ensured the system did not run out of memory (as would happen when using a local computer), allowing all images in the training and test datasets to be stacked into a single tensor.

Before processing the data, all images were renamed to include the subject letter, sample number, class, replica, and image number, which corresponded to the LED excitation and emission filter combination. Then, the saturation information was extracted from each image. Nine images were stacked into a 3D tensor, with one dimension representing the particular excitation-emission combination (i.e., separate autofluorescence images – see Table 1). In contrast, the other dimensions represent the horizontal and vertical spatial directions of each image, as depicted in step 3 of Supplementary Figure S3. Rather than using full images, nine 700 × 700-pixel patches (approximately 14 μm x 14 μm) were extracted from each image to amplify the available training data. Once the dataset was split into training and test sets, image augmentation was applied to the training set by rotating the stacks of image patches as described previously and illustrated in steps four and five of Supplementary Figure S3. These 3D data points, saved as .npz files, were then used as training data for the CNN. Moreover, the .npz files were consolidated into a single data tensor along with a corresponding label tensor for both the training and testing datasets. Based on the image stack name, the image stacks were labeled as 0 or 1 to indicate CoNS (negative) and *S. aureus* + CoNS (positive) mixtures, respectively. All bacterial mixtures (CoNS and *S. aureus* + CoNS mixtures at varied ratios), each with nine patches and two rotations, were randomly split into train and test at an 80:20 ratio, and training models were created for each split. While each image patch is considered independent when the data is split into training and test subsets, all five patches from the same image, including their rotated versions, were placed in the same subset to prevent data leakage between groups. Specifically, a random number generator was used to generate a sequence of four numbers corresponding to the sample numbers, ranging from 1 to 20. The four numbers generated in this sequence were the sample numbers moved into the test dataset, while the remaining samples were placed in the training dataset. Splitting data in this manner ensures that there is no data leakage between groups and that the test dataset is completely unknown. Various hyperparameters were tuned to train the CNN model, including the learning rate schedule, number of filters, kernel size, activation function, optimizer, and dropout layers. The output activation function was a sigmoid function for binary classification (*S. aureus* negative or positive), as depicted in step six of Supplementary Figure S3. A binary cross-entropy loss function was monitored. These hyperparameters were tuned manually through trial and error. After each hyperparameter tuning trial, the learning and loss curves were evaluated to check for underfitting and overfitting. Validation accuracy was the metric used to determine which hyperparameters were the most successful. Note that training the model for each manually tuned hyperparameter took approximately 8 h to complete on the HPC.

To assess the initial feasibility of the device’s application to real-world mixed bacterial samples, human skin swab samples were collected. The human subject samples served as a preliminary test and example of a complex bacterial mixture. Samples were collected from the subject at varied dates and times. They were spiked with CoNS and *S. aureus* (no CoNS was added to the *S. aureus* sample due to the existence of commensal bacteria from human skin swab samples). The CNN analysis was conducted slightly differently with *Staphylococci*-spiked skin swab samples. The value channel was utilized instead of the saturation channel used in the bacterial mixtures. This change was made after analyzing the raw human skin swab sample images in the HSV color space, which revealed greater variations among the data and was confirmed by the CNN analysis. Additionally, the images were cropped into five 500 × 500-pixel patches rather than the nine 700 × 700-pixel patches for the bacterial mixtures. While the decrease in patch number ultimately leads to a smaller dataset, cropped image patches ensure that no overlapping regions are present, thereby reducing the risk of overfitting. Initially, image stacks (each with five patches x four rotations x nine excitation-emission combinations) were randomly split into training and test sets at an 80:20 ratio or were validated using leave-one-out cross-validation (LOOCV). Once again, the splitting was done so that all rotations and patches originating from the same sample number are in the training dataset or the testing dataset. Then, with the LOOCV, each sample served as a test dataset, while the remaining samples were used for training. This process was repeated until all the samples had been held out as the test dataset. The CNN models share a similar architecture to the model created for the bacterial mixtures, with slight adjustments to the hyperparameters, as these were specifically tuned for the skin swab sample models. Hyperparameter tuning for the skin swab samples was performed using Keras Tuner with the RandomSearch algorithm. A single subject sample was randomly selected for the hyperparameter tuning, and the resulting optimal parameters were applied to all subsequent subject samples. The search space was defined to include the number of convolutional layers (2, 3, 4, 5), number of filters per layer (16, 32, 48, 64), kernel size (3, 5, 7), activation function (ReLU, tanh, sigmoid), and dense layer size (64, 128, 192, 256, 320, 384, 448, 512). To identify the optimal hyperparameters, 25 random combinations were evaluated, each trained for 100 epochs. Validation accuracy was used as the performance metric to select the best-performing configuration. The addition of grid search for hyperparameter tuning and the incorporation of LOOCV increased the computational time required to train the model, with the model taking approximately 24 h to run, highlighting the need for HPC.

After the initial CNN models were created and evaluated, attempts were made to improve them by further pre-processing the data before feeding it into the CNN model. Specifically, when images were deemed suboptimal, i.e., blurry or dim, they were removed from the dataset. Since CNNs can handle raw image inputs and are commonly used for image recognition, they can effectively extract morphological features of bacterial structures and clusters, aiding in classification. Therefore, it is crucial that the images fed into the model are well-focused and that the bacterial structures and clusters are clearly visible. Otherwise, the model may become confused or learn irrelevant features, thereby hindering its classification accuracy. The removal of below-par images is automated using an ImageJ macro (Supplementary Code S3). This macro applies a circular mask to isolate the region corresponding to the microscope’s field of view, then calculates the average intensity within this region. Additionally, average sharpness values were collected from all images (five patches x four rotations x nine excitation-emission combinations) of each sample, representing the image’s “blurriness.” These intensity and sharpness values are then exported to a .csv file. When the intensity and sharpness values are graphed, two distinct clusters emerge: one group of data points is clustered around higher average intensity (or sharpness) values, and the other group is centered around lower values. The intensity (or sharpness) value separating these two clusters is the cutoff for eliminating dim (or blurry) images. In addition, since this method is applied to each of the nine LED-filter combination images, which are stacked into a single input tensor, if one of the images in the stack is deemed unusable, the entire image stack is eliminated from the dataset. Such eliminations cannot eliminate the inherent resolution limitations of a smartphone-based microscope, as described in the discussion section. After this additional pre-processing, the CNN models were constructed as previously described.

## Results

### LDA with bacterial mixtures

LDA was conducted using the average saturation values from the areas of interest (i.e., the regions exhibiting autofluorescence) from the bacterial mixtures – namely, *S. aureus* negative (CoNS) and *S. aureus* positive (CoNS + *S. aureus*) bacterial mixtures. It achieved an average sensitivity of 88%, specificity of 29%, F1 score of 76%, and accuracy of 65% from 10-fold cross-validation with three repeats per fold (Table 2). Additionally, the corresponding averaged per-fold confusion matrices are shown in Supplementary Figure S5. Hence, this model was deemed insufficient for binary classification and was not further validated. The results demonstrate the need for a more complex algorithm, such as a deep learning method (e.g., CNN), to analyze morphological features in these images.

**Table 2:** Average accuracy, sensitivity, specificity, and F1-score of the LDA model with bacterial mixtures

Fold	Sensitivity	Specificity	F1	Accuracy
1	0.944 ± 0.096	0.000 ± 0.000	0.722 ± 0.048	0.567 ± 0.058
2	0.889 ± 0.096	0.333 ± 0.289	0.763 ± 0.011	0.667 ± 0.058
3	0.833 ± 0.167	0.250 ± 0.000	0.710 ± 0.092	0.600 ± 0.100
4	0.944 ± 0.096	0.250 ± 0.000	0.771 ± 0.049	0.667 ± 0.058
5	0.944 ± 0.096	0.167 ± 0.144	0.755 ± 0.043	0.633 ± 0.058
6	0.833 ± 0.000	0.417 ± 0.144	0.751 ± 0.032	0.667 ± 0.058
7	0.722 ± 0.255	0.500 ± 0.250	0.689 ± 0.102	0.633 ± 0.058
8	0.889 ± 0.096	0.250 ± 0.250	0.745 ± 0.028	0.633 ± 0.058
9	0.857 ± 0.143	0.444 ± 0.192	0.815 ± 0.054	0.733 ± 0.058
10	0.905 ± 0.082	0.333 ± 0.333	0.827 ± 0.029	0.733 ± 0.058
Average	0.876 ± 0.126	0.294 ± 0.217	0.755 ± 0.062	0.653 ± 0.073

### CNN with bacterial mixtures

**Fig. 3:**
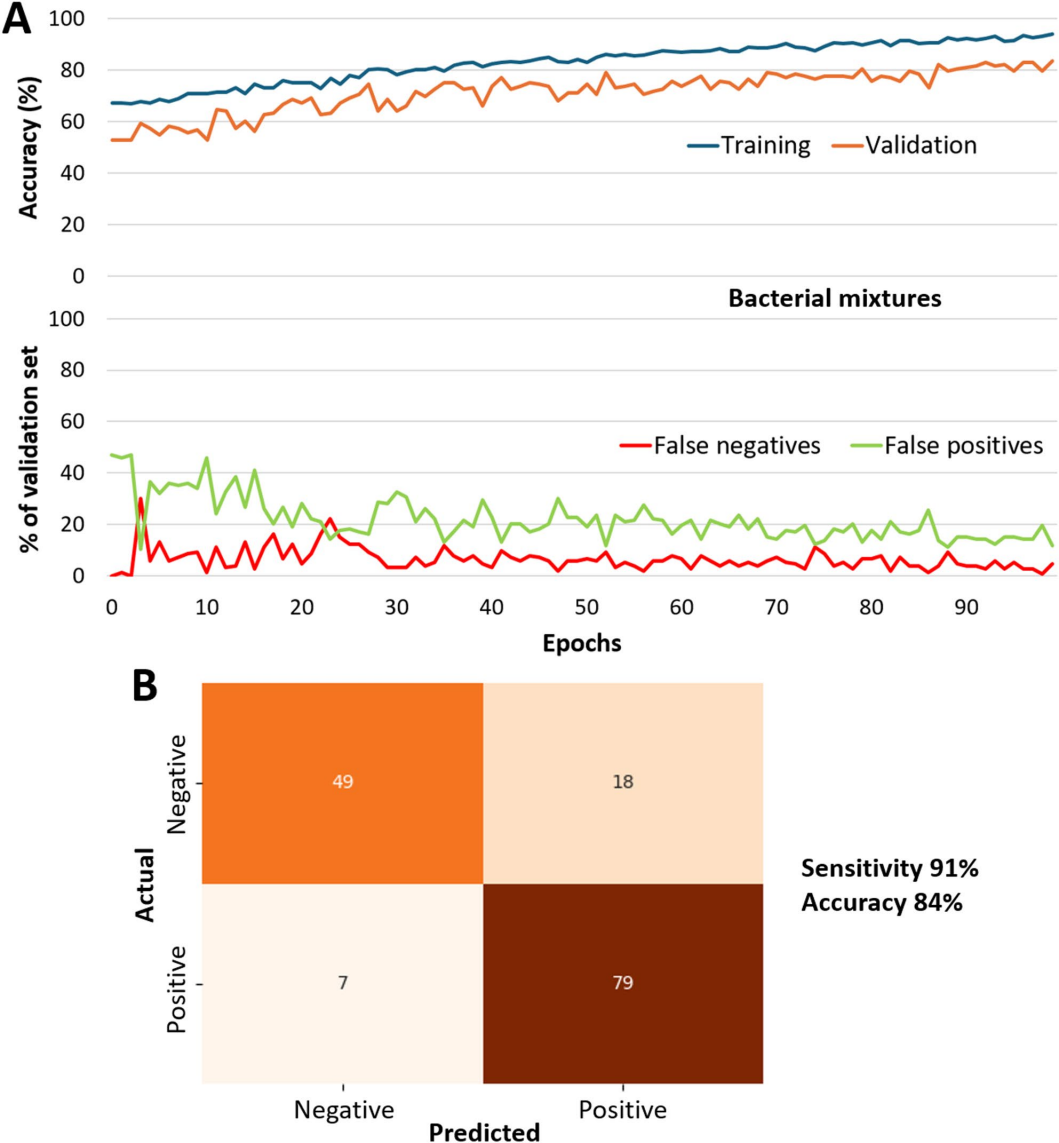
CNN result of bacterial mixtures. (**A**) Representative CNN result. (**B**) Confusion matrix for all samples

The CNN training dataset consisted of 1,445 image stacks from *S. aureus-*negative and positive bacterial mixtures (CoNS vs. CoNS + *S. aureus* with varying ratios). 1,292 were used for training, and 153 were used for validation. The hyperparameters for the highest-performing model in training are shown in Supplementary Table S1. The model’s performance over time during training is shown in Fig. 3. Furthermore, although the test dataset is relatively small, with only 153 samples, the learning curve shown in the top panel of Fig. 3A illustrates similar behavior between the training and validation sets, with steadily increasing accuracy over epochs, thereby confirming the model’s generalizability. Since there is no divergence between the training and validation learning curves, the model is neither underfitting nor overfitting. It demonstrated excellent performance in identifying *S. aureus* presence, with a sensitivity of 91%. However, a small number of false positives compromised the overall accuracy to 84%. The confusion matrix for this model is shown in Fig. 3B. Overall, the CNN model was superior to the LDA, indicating the need for deep learning analysis of morphological features in addition to autofluorescent intensities.

### CNN with skin swab samples

The images collected from skin swab samples differed substantially from those obtained from bacterial mixtures, particularly in autofluorescence intensity. Therefore, a new CNN training model was developed and trained on the image datasets from skin swab samples. We collected human skin swabs at multiple dates and times to ensure maximum data variability within the single human subject. The hyperparameters outlined in Supplementary Table S1 were applied to each model using either a different random split of the dataset or a given leave-one-out cross-validation (LOOCV) set. Initially, 20 samples were collected, and for each sample, three trials of images were acquired. This results in a total of 120 image stacks, or 60 image stacks per class. After image augmentation, the images were cropped into patches, which were then rotated. This resulted in 1,200 image patches per class, totaling 2,400 image patches. The data is split into training and test datasets at a ratio of 80:20. This results in 48 CoNS and 48 *S. aureus* image stacks or 960 image patches per class in the training dataset. As explained in the Materials and Methods, whenever the images were blurry or dim, we removed the entire image set for that sample (5 patches x 4 rotations x 9 excitation-emission combinations) that were deemed suboptimal. After removing suboptimal images, a total of 37 image stacks or 740 image patches remained in the training dataset. Of the 37 total image stacks, 22 were of *S. aureus* while the remaining 15 were of CoNS, resulting in 440 and 300 patches per class, respectively, after augmentation. Additionally, the testing dataset comprises 12 CoNS and 12 *S. aureus* image stacks. Alternatively, there are 240 image patches after augmentation in both the CoNS and *S. aureus* classes in the testing dataset. After removing suboptimal images, 49 image stacks remained out of the initial 120, for a 40% retention rate. Such a retention rate can be attributed to the extreme difficulty in manually focusing the smartphone microscope attachment using complicated skin swab samples.

**Fig. 4:**
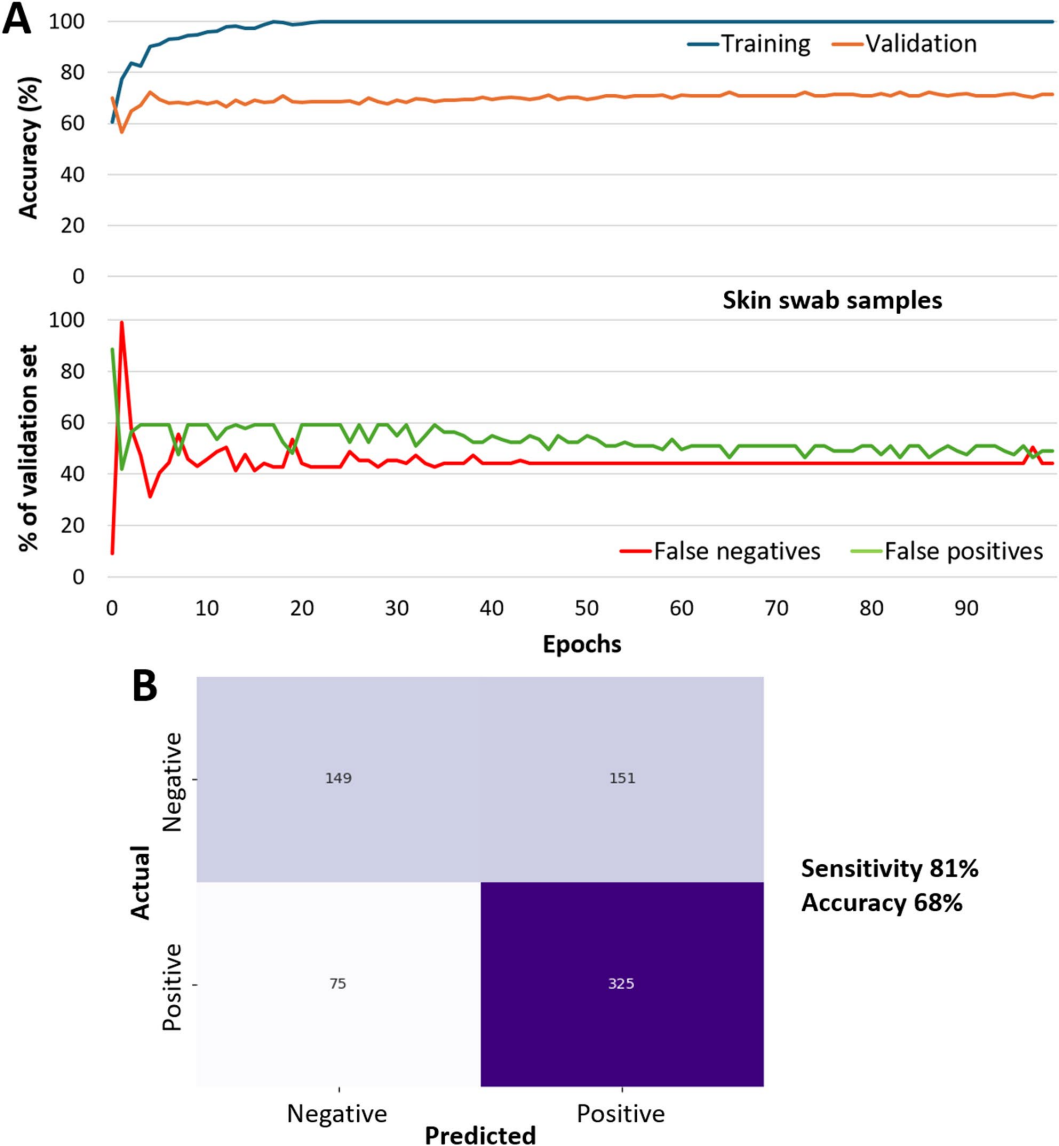
CNN result of skin swab samples. (**A**) Representative CNN result. (**B**) Confusion matrix for all samples

One representative performance of the model during training over time is shown in Fig. 4A, which displays a validation accuracy of 72% for a specific random split of the training and test data sets. The learning curve shows that training accuracy increases and stabilizes toward a high value. While the validation accuracy does not reach the same level as the training accuracy, it shows a gradual and stable increase over the course of the epochs. This stabilization of the validation accuracy indicates that the model is not overfitting, as the smoothness and overall shape of the learning curve are more indicative of the model’s ability to learn and generalize effectively [37].

The confusion matrix representing all samples is shown in Fig. 4B. While the sensitivity (for identifying *S. aureus*) remained satisfactory at 81%, the overall accuracy was compromised to 68% due to the substantial number of false positives. This could be attributed to the inherent limitations of the current smartphone-based device, especially with CoNS-spiked skin swab samples but not *S. aureus*-spiked ones, which makes focusing challenging. Since CNNs can handle raw image inputs and are commonly used for image recognition, they can effectively extract morphological features of bacterial clusters, aiding in classification. Therefore, it is crucial that the images input into the model are well-focused and that the bacterial clusters are clearly visible. Otherwise, the model may become confused or learn irrelevant features, thereby hindering its classification accuracy. While the below-par images were automatically removed using an ImageJ macro (Supplementary Code S3), such removals could not eliminate the inherent resolution limitations of a smartphone-based fluorescence microscope. This variability in performance could be attributed to factors such as age, skin type, the composition of the skin microbiome, and the practice of applying cosmetic products.

Images containing background noise are hypothesized to hinder the CNN model’s performance, as the model may treat background noise as a relevant feature for image classification instead of true autofluorescence. Such background noise is more pronounced when the pathogenic target, *S. aureus*, is absent, resulting in a substantial number of false positives and compromising overall accuracy. In contrast, the sensitivity in detecting *S. aureus* remains satisfactory.

## Discussion

This work presents the first proof-of-concept demonstration of a low-cost, staining-free, bioreceptor-free method for analyzing bacterial mixtures with minimal reliance on laboratory equipment. This method did not require any reagents or staining and relied on inexpensive optical equipment (LEDs, low-cost color films as optical filters, a smartphone, and a smartphone microscope attachment). These results likely represent a baseline that can be further improved, which may require a more advanced microscope attachment or other optical equipment.

Initially, bacterial mixtures of *Staphylococcus* spp. (representing skin microbiome) were prepared, and the simpler LDA model was evaluated using only the color intensities from the areas of interest. It yielded low accuracy (64%). In comparison, the accuracy of the complex CNN model was improved to 84% with a very high sensitivity of 91%. Since the CNN model incorporates morphological features of bacterial structures and clusters, these morphological features are likely to play a key role in distinguishing between negative and positive samples. It is worth noting that the LDA used all nine saturation values as input features, and that feature elimination could slightly improve the accuracy. Nonetheless, we only wanted to verify whether incorporating morphological features using a CNN could improve accuracy, and as confirmed, we proceeded with the CNN without further optimizing the LDA model.

Using this CNN model, we further tested human skin swab samples spiked with the same *Staphylococcus* spp. The accuracy dropped to 68%, while the sensitivity was still satisfactory at 81%. However, the model had a high false-positive rate, resulting in a low specificity of 49%, indicating that it struggled to identify samples that do not contain *S. aureus*. This result suggests a potential need to improve sample preparation procedures, image pre-processing, and machine learning algorithms. Furthermore, after removing suboptimal images, 40% of the original dataset remained due to the difficulty in manually focusing the complex skin swab samples. However, this does not equate to 60% of the individual acquired images being suboptimal. For each sample, nine images corresponding to different LED and filter combinations are stacked into a single multidimensional image stack, which serves as a single input sample for the CNN. The CNN model cannot be trained on inputs with varying shapes, so all image stacks must be consistent in size. Therefore, all nine images in a stack must be usable. Thus, if one of the nine images is suboptimal, the entire stack, including the remaining eight images that are not suboptimal, must be removed.

Initially, the CNN model was attempted to be improved by removing certain features of LED and emission filter combinations from the image stacks per sample. This was achieved by analyzing the raw images and removing any LED filter combination that yielded mostly dark images with little to no fluorescent signals. However, this resulted in a lower accuracy of the CNN model. Moreover, since CNN cannot directly determine which image features primarily influence classification accuracy, the model could have benefited from feature elimination based on the feature importance ranking from the LDA results. However, the current work did not attempt it, as the LDA model did not utilize morphological features and its accuracy was substantially lower than that of the CNN model. Additionally, since the bacterial mixtures were tested on a small validation dataset and the CNN model does not appear to be underfitting or overfitting, it could benefit from further validation methods such as 10-fold cross-validation or LOOCV. The transparency of CNN decision-making is limited, as CNNs extract features for classification from images by performing multiple convolutional layers. However, the model does not indicate which image features were used for classification; therefore, it is difficult to determine which morphological features of bacterial autofluorescence were used in classification. Thus, performing interpretability analysis such as gradient-weighted class activation mapping (Grad-CAM) would offer more insight. However, such an analysis was not conducted in the current work, as it falls outside the scope of this feasibility study of a low-cost device. Future developments of the CNN model would benefit from Grad-CAM, which provides more insight into which parts of the image the model considers most important for classification. This analysis would provide further validation that the autofluorescence from bacterial clusters is being used to make predictions rather than background noise in the image.

Future work could also test the model with different total bacterial concentrations. In this study, the overall bacterial load in both the prepared mixtures and the clinical samples was kept approximately constant, allowing us to specifically evaluate the model’s ability to distinguish the most prevalent bacterial species within each sample. Testing the model with different concentrations of bacteria could help identify an optimal concentration range for which this methodology is applicable and determine whether exposure and other camera settings need to be optimized for different concentrations.

Since the low-cost microscope attachment does not provide sufficient resolution to distinguish between different shapes of bacterial clusters (especially among the *Staphylococcus* species), accurate classification is currently limited by the available low-cost equipment used in this study. Using a higher-resolution microscope would enable the incorporation of more nuanced morphological characteristics of the bacteria into the CNN model, such as size, shape, and spatial arrangement of bacterial structures and clusters. In addition, focusing is challenging with the current smartphone-based microscope system, as the image remains unfocused, despite adjustments to the focus knob. The focal length should have been adjusted for each image and each LED-filter combination, which is constrained by the very close working distance between the microscope glass slide and the microscope attachment. It is essentially an upright setting rather than an inverted setting. In addition, focusing is even more challenging when imaging skin swab samples, as they generate non-specific autofluorescence from dead cells, skin pigments, fabric particles, skin care products, and lipids. The skin swab samples are more complex than the bacterial mixtures, as they contain greater variability in bacterial species represented in the prepared mixtures. These samples also contain more background noise from dead skin cells and other debris. These factors likely contributed to increased noise in the images and were one of the reasons model performance decreased on the clinical samples. Thus, the model might improve with more refined sample processing before imaging to remove this background noise and isolate only the bacteria obtained from the skin swab.

While standard benchtop fluorescence microscopy of the bacteria mixtures and skin swab samples might help evaluate this method’s ability to distinguish dominate bacteria species independently of the quality of the optical system, this work did not verify the results with this approach since the goal was to develop a low-cost, portable, image-based approach that does not rely on traditional benchtop equipment or laboratory-based microbiological assays and culturing. Additionally, the low-cost color films, another important aspect of this work, cannot be installed on a benchtop fluorescence microscope, and the resulting machine learning classification would change significantly. Nonetheless, future work could compare the results of this low-cost approach with those of standard fluorescence microscopy to confirm its robustness and accuracy.

Additionally, some images captured with the device remained extremely dim regardless of the exposure time set on the camera, making it difficult to see the autofluorescence signal. This could suggest that a stronger light source is needed to excite the fluorophores or that the camera is not sensitive enough to collect the signal. It is worth noting that during device construction, the radiant power of the LEDs was not measured to quantify the light intensity reaching the sample, nor was it matched to the smartphone’s camera sensor to ensure no oversaturation of the images. However, this would be an important consideration for future versions of the device, allowing for more consistent data.

In addition to obtaining higher-resolution images, the device could also be improved to include automatic image uploading to a cloud server and an easy-to-use graphical user interface on a smartphone, allowing users to capture images, run the CNN code on the cloud, and display the results on the smartphone screen. Along these lines, it is worth noting that while the HPC was used and necessary for the machine learning models, this might not be the case in certain situations. If a model had already been trained and the user was testing it on a new sample or a small independent validation set, it could run on a local computer or a commercial cloud server connected wirelessly to a smartphone. Nonetheless, we believe HPC remains necessary for building a learning database and optimizing a machine learning algorithm, which was the case for this study.

Another challenging aspect of this project is the classification that attempts to distinguish between bacterial mixtures composed of bacteria within the same genus, *Staphylococcus*. Bacterial species are similar in morphology and may exhibit similar spectral signals and thus, similar autofluorescence characteristics. This similarity makes it challenging to differentiate between the two groups. It would be less difficult to distinguish samples containing different bacterial genera.

This work provides a proof-of-concept for an alternative method to identify bacterial species in mixed samples using a low-cost smartphone-based imaging system. In this work, a machine learning model was built using laboratory-simulated samples before moving to more complex real-world samples, such as skin swab samples. To make this method more robust, further testing is required to evaluate the device with more complicated and comprehensive bacterial mixtures, as well as to test it on a larger cohort of human subjects.

## Conclusion

This work presents a novel, non-invasive, inexpensive, and portable method and platform for classifying bacterial mixture samples as either *S. aureus*-negative or *S. aureus*-positive. It used a smartphone, a set of low-cost color films, and a low-cost smartphone microscope attachment to capture a large set of autofluorescent images from laboratory-prepared bacterial mixtures and skin swab samples spiked with *S. aureus*. This method can detect the presence of *S. aureus* at levels that naturally occur in the skin microbiome with approximately 10^4^ bacteria per cm^2^. Using the CNN model, the method achieved 84% accuracy and a 91% sensitivity for bacterial mixture (*Staphylococcus* spp.) samples. The same model for the more complex skin swab samples yielded compromised accuracy of 68%, while sensitivity remained satisfactory at 81%, using leave-one-out cross-validation (LOOCV), presumably due to the skin swab samples’ greater complexity. Improved results could be achieved with a higher-resolution smartphone microscope attachment featuring a more robust focusing mechanism and a much stronger light source.

## Supplementary Information

Below is the link to the electronic supplementary material.

Supplementary Material 1

## Data Availability

Additional data and codes are available in the Supplementary Information. Further data can be requested from the authors upon reasonable request.
