# Supplementary material for: Smartphone-based multispectral autofluorescence analysis of bacteria mixtures of staphylococci using convolutional neural network
Source: J Biol Eng. 2026 Jan 23;20:15. doi: 10.1186/s13036-025-00616-7 (PMC12836997; doi:10.1186/s13036-025-00616-7)
Supplement: Supplementary file 1 — Supplementary Material 1 [file 13036_2025_616_MOESM1_ESM.pdf]

Supplementary Materials

Smartphone-Based Multispectral Autofluorescence Analysis of Bacteria Mixtures of Staphylococci Using Convolutional Neural Network

Jocelyn Reynolds, Katelyn Sosnowski, Christine Carlson, Thomas D. McGuire, Will Roman, and Jeong-Yeol Yoon

Department of Biomedical Engineering, The University of Arizona, Tucson, Arizona 85721, USA

Supplementary Figure S1. Spectra of excitation LEDs.

Supplementary Figure S2. Magnified versions of Figure 2.

Supplementary Figure S3. Overview of experimental and machine learning classification procedures.

Supplementary Figure S4. Sample image areas that cannot be analyzed for average saturation.

Supplementary Figure S5. Fold-averaged confusion matrices from 10-fold cross-validation of the LDA.

Supplementary Table S1. CNN hyperparameters.

Supplementary Code S1. LDA code.

Supplementary Code S2. CNN code.

Supplementary Code S3. ImageJ macro.

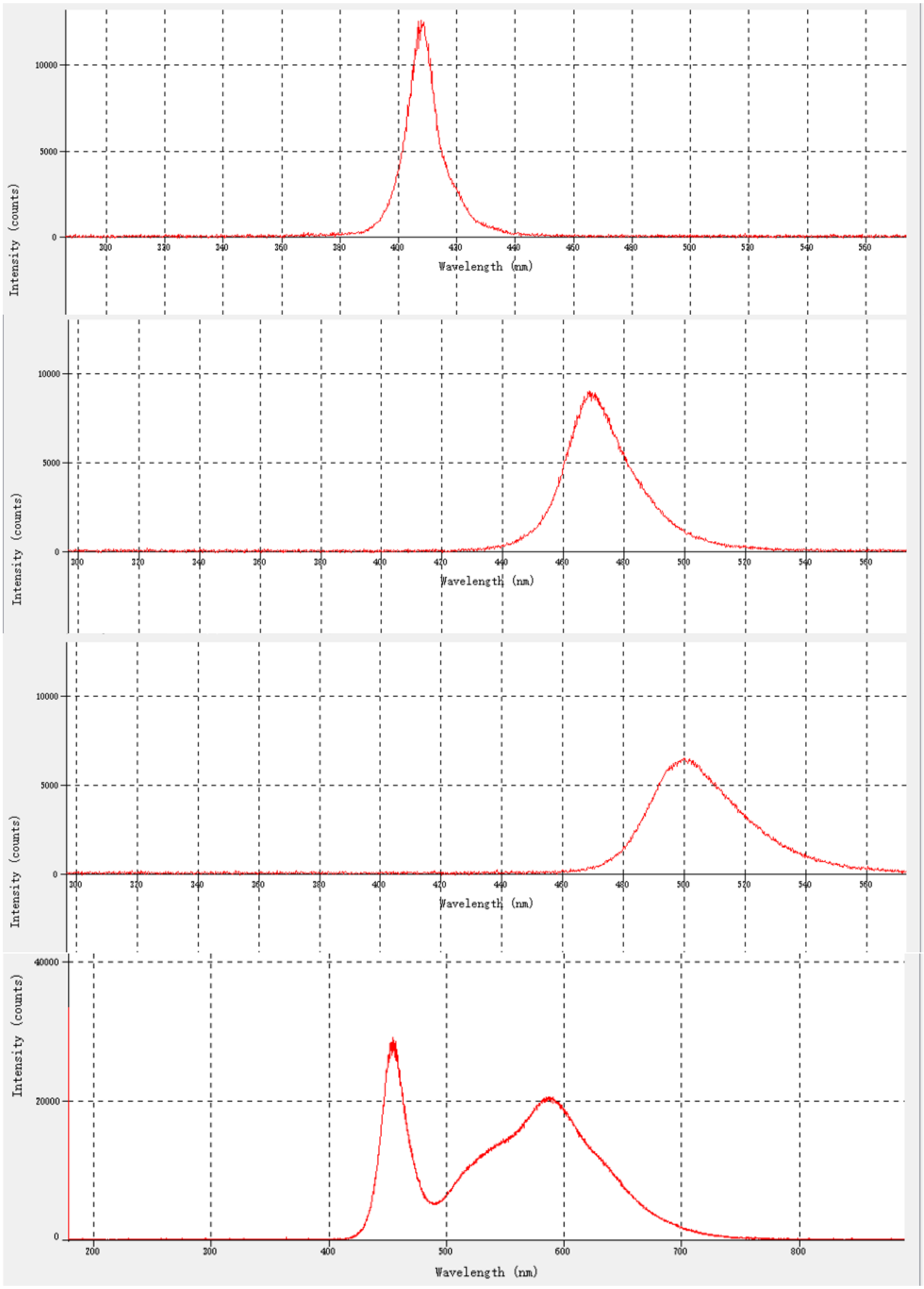

Supplementary Figure S1. Spectra of excitation LEDs. From top to bottom: 405 nm, 460 nm, 489 nm, and a white LED.

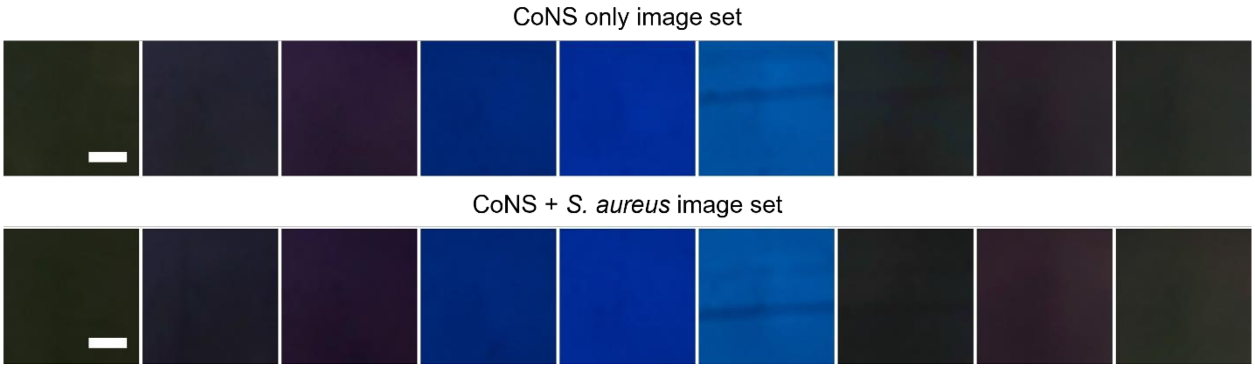

Supplementary Figure S2. Magnified versions of Figure 2. Scale bar = 200 µm.

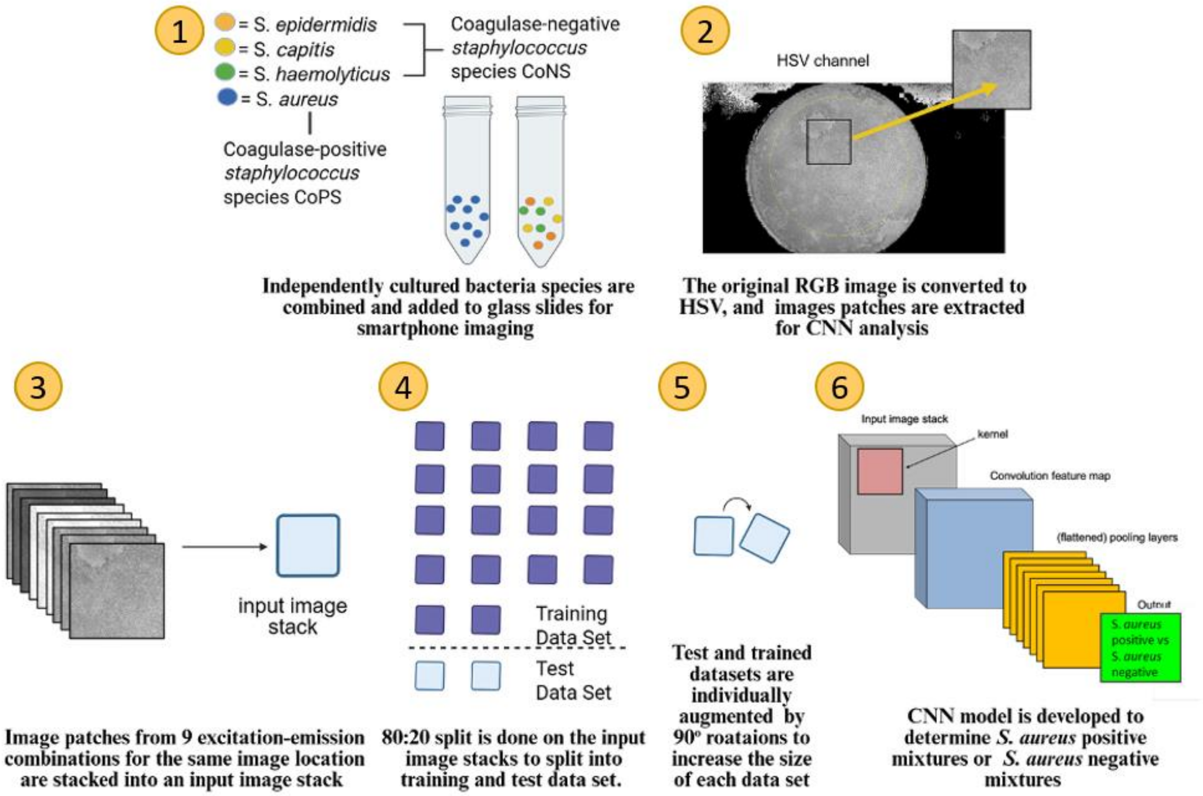

Supplementary Figure S3. Overview of experimental and machine learning classification procedures.

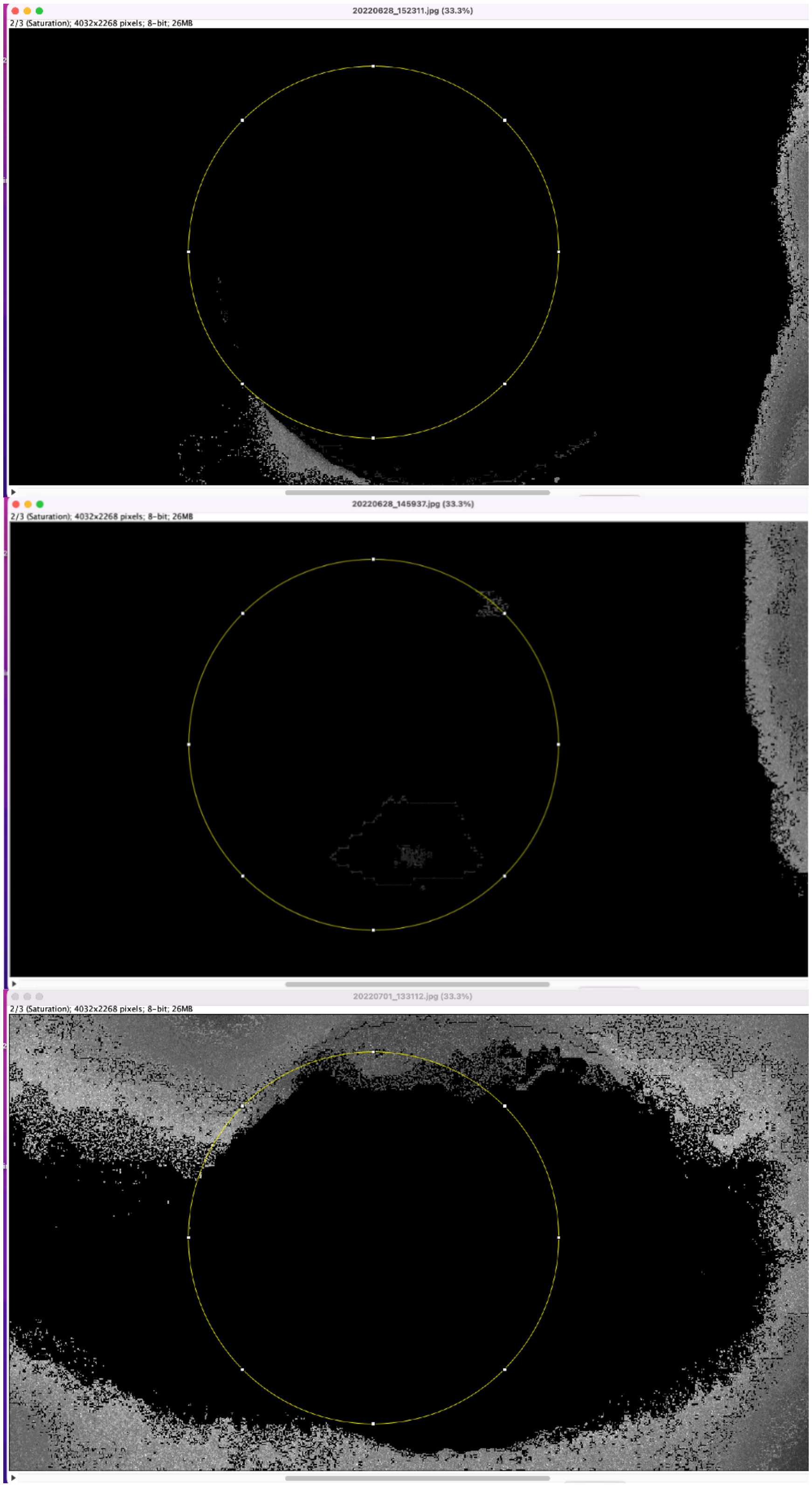

Supplementary Figure S4. Sample image areas that cannot be analyzed for average saturation. These areas were given the value –1 instead of an average saturation.

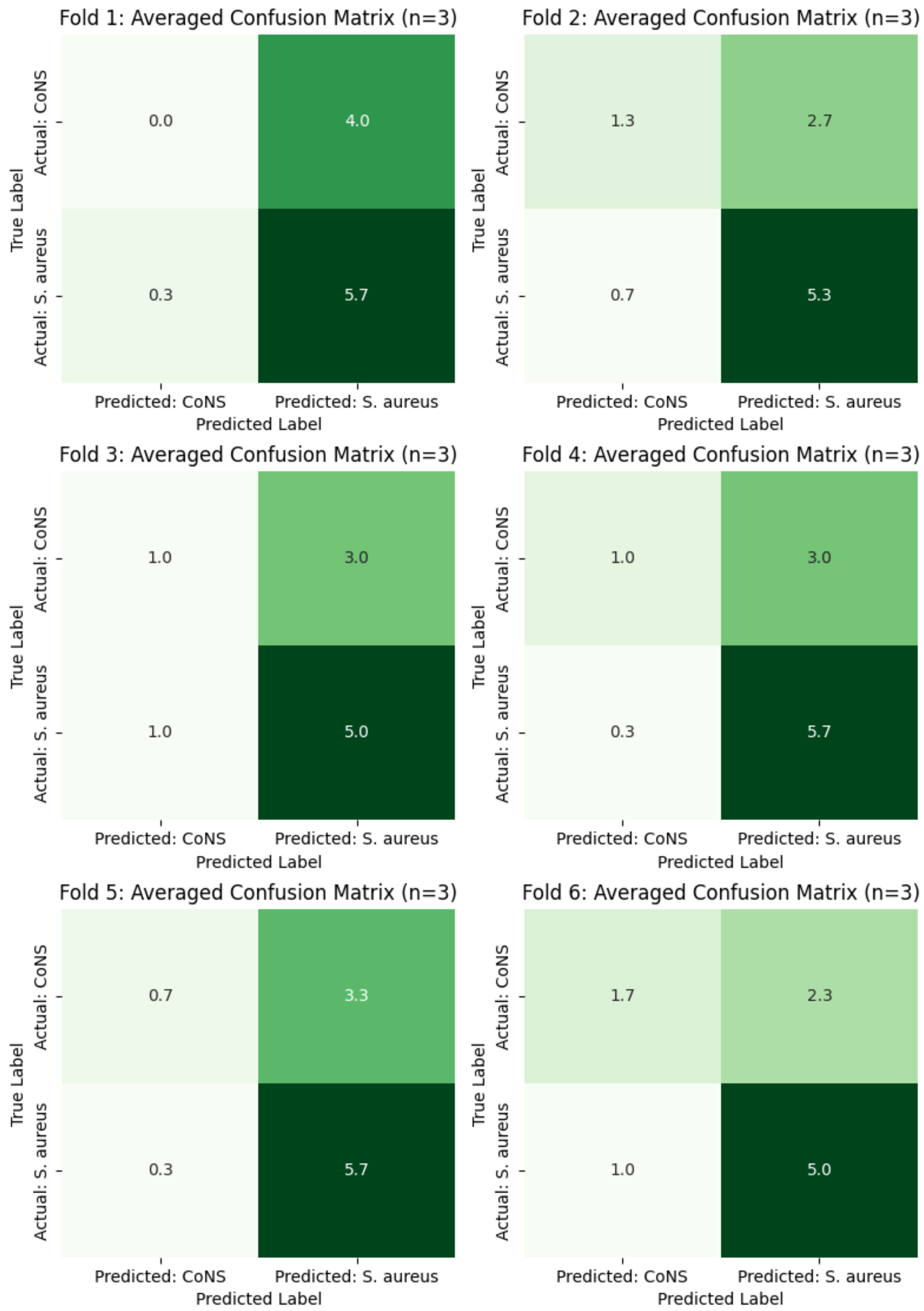

Supplementary Figure S5. Fold-averaged confusion matrices from 10-fold cross-validation of the LDA. Each subplot represents the average confusion matrix for one fold (each consisting of three trials), illustrating the classification performance between CoNS and *S. aureus* + CoNS samples.

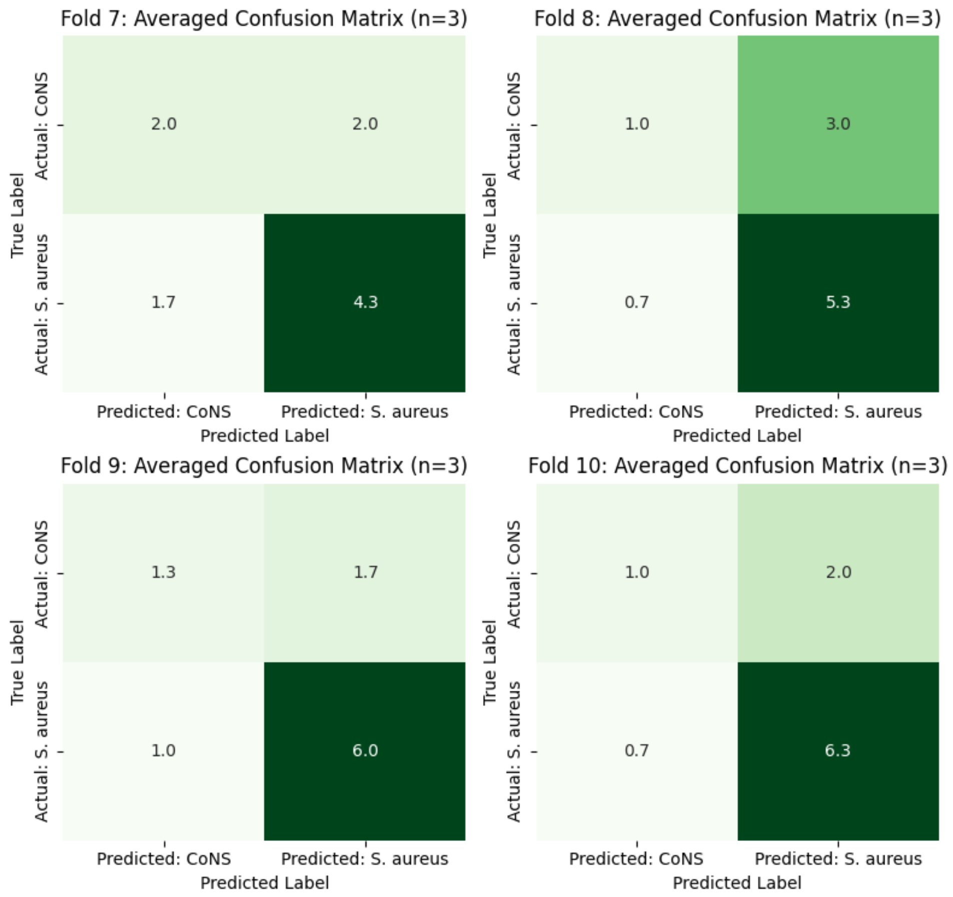

Supplementary Figure S5. Fold-averaged confusion matrices from 10-fold cross-validation of the LDA (continued).

Supplementary Table S1. CNN hyperparameters.

Parameter	Bacterial mixtures	Skin swab samples
Num conv layers	3	4
Filters	20, 15, 10	32, 16, 16, 16
Kernel size	7 × 7	5 × 5
Activation function	Relu	Relu
Pooling type<br>Size	MaxPooling2D<br>25 × 25	MaxPooling2D<br>5 × 5
Output layer	Dense(2), Sigmoid	Dense(1), Sigmoid
Learning rate	0.001 with Inverse Time Deca	0.001 (Adam optimizer default)
Batch size	1	32
Epochs	15	100
Learning rate	Inverse time decay<br>decay rate: 0.9, steps: 10,000	None

Supplementary Code S1. LDA code.

```
"""
Skin Microbiome Autofluorescence Project

Authors: Katie Sosnowski and Christine Carlson

See https://scikit-
learn.org/stable/modules/generated/sklearn.discriminant_analysis.LinearDiscriminantAnalysis.html
and https://www.statology.org/linear-discriminant-analysis-in-python/
"""

#Imports
import numpy as np
import pandas as pd
import matplotlib.pyplot as plt
from sklearn.discriminant_analysis import LinearDiscriminantAnalysis
from sklearn.model_selection import train_test_split, RepeatedStratifiedKFold, cross_val_score
from sklearn import metrics

#Load dataset
#Unclear why everything had to be hard-coded, but it works
train_file =
pd.DataFrame(pd.read_csv('data_imagej_sat.csv'),columns=["bac_date_rep","label","405_10","405_12","
405_15","460_15","460_20","460_2004","405_2004","460_22","489_22"])
train_labels = list(train_file.loc[0:99,'label'])
train_data = train_file.drop(['bac_date_rep', 'label'], axis=1)
train_data=train_data.loc[0:99,:]

#Define and fit LDA model
clf = LinearDiscriminantAnalysis()
clf.fit(train_data, train_labels)

#Stratified K-fold cross-validation
cv=RepeatedStratifiedKFold(n_splits=10, n_repeats=3)
scores=cross_val_score(clf, train_data, train_labels, scoring='accuracy', cv=cv)
print(np.mean(scores))
```

Supplementary Code S2. CNN code.

Image Stacking:

```
"""
Skin Microbiome Research Project: Image Stacking Code (image patches)

Author: Katie Sosnowski

Info about NPZ files: https://machinelearningmastery.com/how-to-save-a-numpy-array-to-file-for-
machine-learning/
"""

# Utility libraries
import sys
import os
# OpenCV
#import cv2
# Numpy and math
import numpy as np
from numpy import savez_compressed #for saving NPZ files
import math

# Matplotlib for visualization
import matplotlib.pyplot as plt
from matplotlib import image
#from matplotlib import pyplot

# skimage
from skimage import data
from skimage.color import rgb2hsv

# Define pathways for image sets
#Training sets
#bacteria_sets =['2022-06-27/CoNS/rep1', '2022-06-27/CoNS/rep2','2022-06-27/CoNS/rep3','2022-06-
27/5050/rep1', '2022-06-27/5050/rep2','2022-06-27/5050/rep3', '2022-06-27/7030/rep1', '2022-06-
27/7030/rep2','2022-06-27/7030/rep3', '2022-06-28/CoNS/rep1', '2022-06-28/CoNS/rep2','2022-06-
28/CoNS/rep3', '2022-06-28/CoNS/rep4', '2022-06-28/CoNS/rep5','2022-06-28/CoNS/rep6','2022-06-
28/5050/rep1', '2022-06-28/5050/rep2','2022-06-28/5050/rep3', '2022-06-28/5050/rep4', '2022-06-
28/5050/rep5','2022-06-28/5050/rep6','2022-06-28/7030/rep1', '2022-06-28/7030/rep2','2022-06-
28/7030/rep3', '2022-06-28/7030/rep4', '2022-06-28/7030/rep5','2022-06-28/7030/rep6', '2022-07-
01/CoNS/rep1', '2022-07-01/CoNS/rep2', '2022-07-01/CoNS/rep3', '2022-07-01/5050/rep1', '2022-07-
01/5050/rep2', '2022-07-01/5050/rep3', '2022-07-01/7030/rep1', '2022-07-01/7030/rep2', '2022-07-
01/7030/rep3', '2022-07-07/CoNS/rep1', '2022-07-07/CoNS/rep2', '2022-07-07/CoNS/rep3', '2022-07-
07/CoNS/rep4', '2022-07-07/CoNS/rep5', '2022-07-07/CoNS/rep6', '2022-07-07/CoNS/rep7', '2022-07-
07/CoNS/rep8', '2022-07-07/CoNS/rep9', '2022-07-07/5050/rep2', '2022-07-07/5050/rep3', '2022-07-
07/5050/rep4', '2022-07-07/5050/rep5', '2022-07-07/5050/rep6', '2022-07-07/5050/rep7', '2022-07-
07/5050/rep8', '2022-07-07/5050/rep9', '2022-07-07/7030/rep3', '2022-07-07/7030/rep4', '2022-07-
07/7030/rep5', '2022-07-07/7030/rep6', '2022-07-07/7030/rep7', '2022-07-07/7030/rep8', '2022-07-
07/7030/rep9', '2022-07-14/CoNS/rep1', '2022-07-14/CoNS/rep2', '2022-07-14/CoNS/rep3', '2022-07-
14/CoNS/rep4', '2022-07-14/CoNS/rep5', '2022-07-14/CoNS/rep6', '2022-07-14/5050/rep1', '2022-07-
14/5050/rep2', '2022-07-14/5050/rep3', '2022-07-14/7030/rep1', '2022-07-14/7030/rep2', '2022-07-
```

14/7030/rep3', '2022-09-01/CoNS/rep1', '2022-09-01/CoNS/rep2','2022-09-01/CoNS/rep3', '2022-09-01/5050/rep1', '2022-09-01/5050/rep3', '2022-09-01/7030/rep1', '2022-09-01/7030/rep2', '2022-09-01/7030/rep3', '2022-09-06/CoNS/rep1', '2022-09-06/CoNS/rep3', '2022-09-13/CoNS/rep1', '2022-09-13/CoNS/rep2','2022-09-13/CoNS/rep3','2022-09-13/CoNS/rep4','2022-09-13/CoNS/rep5','2022-09-13/CoNS/rep6','2022-09-13/5050/rep1', '2022-09-13/5050/rep2','2022-09-13/5050/rep3','2022-09-13/5050/rep4','2022-09-13/5050/rep5','2022-09-13/5050/rep6', '2022-09-13/7030/rep1', '2022-09-13/7030/rep2','2022-09-13/7030/rep3','2022-09-13/7030/rep4','2022-09-13/7030/rep5','2022-09-13/7030/rep6']

```
#Laboratory holdout set
#bacteria_sets = ['2022-09-20_holdout/5050/rep1', '2022-09-20_holdout/5050/rep2', '2022-09-20_holdout/5050/rep3', '2022-09-20_holdout/5050/rep4', '2022-09-20_holdout/5050/rep5', '2022-09-20_holdout/5050/rep6', '2022-09-20_holdout/5050/rep7', '2022-09-20_holdout/5050/rep8', '2022-09-20_holdout/5050/rep9', '2022-09-20_holdout/7030/rep1', '2022-09-20_holdout/7030/rep2', '2022-09-20_holdout/7030/rep3', '2022-09-20_holdout/7030/rep4', '2022-09-20_holdout/7030/rep5', '2022-09-20_holdout/7030/rep6', '2022-09-20_holdout/7030/rep7', '2022-09-20_holdout/7030/rep8', '2022-09-20_holdout/7030/rep9', '2022-09-20_holdout/CoNS/rep1', '2022-09-20_holdout/CoNS/rep2', '2022-09-20_holdout/CoNS/rep3', '2022-09-20_holdout/CoNS/rep4', '2022-09-20_holdout/CoNS/rep5', '2022-09-20_holdout/CoNS/rep6', '2022-09-20_holdout/CoNS/rep7', '2022-09-20_holdout/CoNS/rep8', '2022-09-20_holdout/CoNS/rep9']

#C. striatum holdout set
bacteria_sets = ['Cstri/CoNS_5050_Cstri/rep1', 'Cstri/CoNS_5050_Cstri/rep2',
'Cstri/CoNS_5050_Cstri/rep3', 'Cstri/5050_Saur_Cstri/rep1', 'Cstri/5050_Saur_Cstri/rep2',
'Cstri/7030_Saur_Cstri/rep1', 'Cstri/7030_Saur_Cstri/rep2', 'Cstri/7030_Saur_Cstri/rep3']

#Human subjects sets
#bacteria_sets = ['human_subjects/002/CoNS/rep1', 'human_subjects/002/CoNS/rep2',
'human_subjects/002/CoNS/rep3', 'human_subjects/002/Saur/rep1', 'human_subjects/002/Saur/rep2',
'human_subjects/002/Saur/rep3', 'human_subjects/003/CoNS/rep1', 'human_subjects/003/CoNS/rep2',
'human_subjects/003/CoNS/rep3', 'human_subjects/003/Saur/rep1', 'human_subjects/003/Saur/rep2',
'human_subjects/003/Saur/rep3', 'human_subjects/004/CoNS/rep1', 'human_subjects/004/CoNS/rep2',
'human_subjects/004/CoNS/rep3', 'human_subjects/004/Saur/rep1', 'human_subjects/004/Saur/rep2',
'human_subjects/004/Saur/rep3', 'human_subjects/005/CoNS/rep1', 'human_subjects/005/CoNS/rep2',
'human_subjects/005/CoNS/rep3', 'human_subjects/005/Saur/rep1', 'human_subjects/005/Saur/rep2',
'human_subjects/005/Saur/rep3', 'human_subjects/006/CoNS/rep1', 'human_subjects/006/CoNS/rep2',
'human_subjects/006/CoNS/rep3', 'human_subjects/006/Saur/rep1', 'human_subjects/006/Saur/rep2',
'human_subjects/006/Saur/rep3', 'human_subjects/007/CoNS/rep1', 'human_subjects/007/CoNS/rep2',
'human_subjects/007/CoNS/rep3', 'human_subjects/007/Saur/rep1', 'human_subjects/007/Saur/rep2',
'human_subjects/007/Saur/rep3', 'human_subjects/008/CoNS/rep1', 'human_subjects/008/CoNS/rep2',
'human_subjects/008/CoNS/rep3', 'human_subjects/008/Saur/rep1', 'human_subjects/008/Saur/rep2',
'human_subjects/008/Saur/rep3', 'human_subjects/009/CoNS/rep1', 'human_subjects/009/CoNS/rep2',
'human_subjects/009/CoNS/rep3', 'human_subjects/009/Saur/rep1', 'human_subjects/009/Saur/rep2',
'human_subjects/009/Saur/rep3', 'human_subjects/010/CoNS/rep1', 'human_subjects/010/CoNS/rep2',
'human_subjects/010/CoNS/rep3', 'human_subjects/010/Saur/rep1', 'human_subjects/010/Saur/rep2',
'human_subjects/010/Saur/rep3']

# Iterate through image sets (folders)
for next_folder in sorted(bacteria_sets):
    # Create a name for the file that has underscores instead of slashes for saving the .npz file
        # And rearrange for easier CNN labeling (bacteria mixture type first)
```

```
		newname = ""
		for char in next_folder:
			if char == '/':
				newchar = '_'
			else:
				newchar = char
			newname += newchar
		newname = newname[6:10] + '_' + newname[0:5] + '_' + newname[22:26]
		#print('date: ', newname[0:10], 'bacteria: ', newname[11:15], 'rep: ', newname[16:20])

	# Iterate through images in the set
		for count, img in enumerate(sorted(os.listdir(next_folder))):
			if not img.startswith('.'): #ignore hidden files
				original = image.imread(next_folder + '/' + img)
				if count==0:
		  # Get dimensions of first image (all images in folder will have same dimension)
					width, height, color = original.shape
					#print('width: ', width, 'height: ', height)
		# Make numpy array of current image
				np_image = np.array(original)
		# Extract SATURATION instead of hue
				hsv_img = rgb2hsv(np_image)
				sat_img = hsv_img[:, :, 1]
		#print('5555')
				if count==0:
		  # Set image volume to the first (croppped) SATURATION image
					image_volume1 = sat_img[457:1179, 1085:1807]
					image_volume2 = sat_img[457:1179, 1807:2529]
					image_volume3 = sat_img[1179:1901, 1085:1807]
					image_volume4 = sat_img[1179:1901, 1807:2529]
					image_volume5 = sat_img[96:818, 1446:2168]
					image_volume6 = sat_img[818:1540, 1446:2168]
					image_volume7 = sat_img[1540:2262, 1446:2168]
					image_volume8 = sat_img[818:1540, 724:1446]
					image_volume9 = sat_img[818:1540, 2168:2890]
				else:
		  # Add (cropped) SATURATION image to existing image volume
					image_volume1 = np.dstack((image_volume1, sat_img[457:1179,
1085:1807]))
					image_volume2 = np.dstack((image_volume2, sat_img[457:1179,
1807:2529]))
					image_volume3 = np.dstack((image_volume3, sat_img[1179:1901,
1085:1807]))
					image_volume4 = np.dstack((image_volume4, sat_img[1179:1901,
1807:2529]))
					image_volume5 = np.dstack((image_volume5, sat_img[96:818,
1446:2168]))
					image_volume6 = np.dstack((image_volume6, sat_img[818:1540,
1446:2168]))
					image_volume7 = np.dstack((image_volume7, sat_img[1540:2262,
1446:2168]))
```

```
                                        image_volume8 = np.dstack((image_volume8, sat_img[818:1540,
724:1446]))
                                        image_volume9 = np.dstack((image_volume9, sat_img[818:1540,
2168:2890]))
                              #print('image volume dimensions: ', image_volume.shape)
   # Save the .npz file in the training database
          savez_compressed('Cstri_sat/' + newname + 'sat_1', image_volume1)
          savez_compressed('Cstri_sat/' + newname + 'sat_2', image_volume2)
          savez_compressed('Cstri_sat/' + newname + 'sat_3', image_volume3)
          savez_compressed('Cstri_sat/' + newname + 'sat_4', image_volume4)
          savez_compressed('Cstri_sat/' + newname + 'sat_5', image_volume5)
          savez_compressed('Cstri_sat/' + newname + 'sat_6', image_volume6)
          savez_compressed('Cstri_sat/' + newname + 'sat_7', image_volume7)
          savez_compressed('Cstri_sat/' + newname + 'sat_8', image_volume8)
          savez_compressed('Cstri_sat/' + newname + 'sat_9', image_volume9)
```

Image Augmentation:

```
"""
Skin Microbiome Research Project: Image augmentation to create more data

Author: Katie Sosnowski

"""

# Utility libraries
import os
import glob

# Numpy and scipy
import numpy as np
from numpy import savez_compressed
from scipy import ndimage


#Go to data folder
os.chdir('human_filtered_sat_train')

for file in sorted(glob.glob('*.npz')):
          npz = np.load(file)
          array = npz['arr_0.npy']
          rotated90 = ndimage.rotate(array, 90)
          rotated180 = ndimage.rotate(array, 180)
          rotated270 = ndimage.rotate(array, 270)
          savez_compressed(file[0:18] + '_rot90', rotated90)
          savez_compressed(file[0:18] + '_rot180', rotated180)
          savez_compressed(file[0:18] + '_rot270', rotated270)
```

Making Tensors:

```
"""
Skin Microbiome Autofluorescence Project
Author: Katie Sosnowski
```

This script is for making tensors out of the test and train data that are saved as zipped files which can be loaded by the CNN script. This way the CNN script does not have to remake these test and train tensors every time the code is re-run for CNN hyperparameter optimization.

```
"""

# Machine learning libraries
import tensorflow as tf
from tensorflow.keras import datasets, layers, models
# Utility libraries
import sys
import os
import glob
# Numpy and math
import numpy as np
from numpy import savez_compressed #for saving NPZ files

# Function for labeling: 0 = CoNS, 1 = S. aureus + CoNS
def labelMaker(filename):
        label = filename[0:4]
        if label=='CoNS':
                label = 0
        elif label=='5050':
                label = 1
        elif label=='7030':
                label = 1
        else:
                label = 'error'
        return label

#Define train dataset
os.chdir('train/')

train_data = []
train_labels = []

# Iterate through the training files
for file in glob.glob('*.npz'):
    #print(file)
    # Fill the list of labels (y data)
    train_labels.append(labelMaker(file))
    # Fill the list of tensors (x data; NPZ files of stacked images)
    with np.load(file) as data:
        train_data.append(tf.convert_to_tensor(data['arr_0']))
# Convert image data into a ragged (unequal image dimensions) tensor
train_data = tf.ragged.stack([array for array in train_data])
# For running the CNN, the ragged tensor needs to then be converted into a tensor
train_data = train_data.to_tensor()
# Re-format the labels list as an array with an extra dimension for batch size
train_labels = np.asarray(train_labels).astype('float32').reshape((-1,1))
# Save train data
```

```
savez_compressed('train_data.npz', train_data)
savez_compressed('train_labels.npz', train_labels)

#Define test dataset
os.chdir('..')
os.chdir('test/')

# Same process for the test data
test_data = []
test_labels = []
for file in glob.glob('*.npz'):
    test_labels.append(labelMaker(file))
    with np.load(file) as data:
        test_data.append(tf.convert_to_tensor(data['arr_0']))
test_data = tf.ragged.stack([array for array in test_data])
test_data = test_data.to_tensor()
test_labels = np.asarray(test_labels).astype('float32').reshape((-1,1))
# Save test data
savez_compressed('test_data.npz', test_data)
savez_compressed('test_labels.npz', test_labels)
```

CNN Model:

```
"""
Skin Microbiome Autofluorescence Project
Author: Katie Sosnowski

This code is for optimizing a CNN to determine whether hue image stacks represent healthy (CoNS-only)
or dysbiotic (S. aureus-overgrown) bacteria samples.

See https://www.tensorflow.org/tutorials/images/cnn for Python Keras TensorFlow CNN tutorial """

# Machine learning libraries
import tensorflow as tf
from tensorflow.keras.optimizers import SGD
from tensorflow.keras import datasets, layers, models
# Utility libraries
import sys
import os
import glob
# Numpy and math
import numpy as np
import math
# Matplotlib for visualization
import matplotlib.pyplot as plt
from matplotlib import image
from matplotlib import pyplot
#skimage
from skimage import data
from skimage.color import rgb2hsv
#pandas
import pandas as pd
```

```
#Load train dataset
with np.load('train/train_labels.npz') as train_labels:
	train_labels = train_labels['arr_0.npy']
with np.load('train/train_data.npz') as train_data:
	train_data = train_data['arr_0.npy']

#Load test dataset
with np.load('test/test_labels.npz') as test_labels:
	test_labels = test_labels['arr_0.npy']
with np.load('test/test_data.npz') as test_data:
	test_data = test_data['arr_0.npy']

#Create a CNN model with alternating CNN and maxpool layers
model = models.Sequential()
#Convolution layer
model.add(layers.Conv2D(10, (7,7), padding = 'same', data_format = 'channels_last', activation='relu',
input_shape=(None, None, 9)))
#Max Pooling layer
model.add(layers.MaxPool2D(pool_size=(25,25), padding='same'))
#Convolution layer
model.add(layers.Conv2D(10, (7,7), padding = 'same', data_format = 'channels_last', activation='relu',
input_shape=(None, None, 9)))
#Add a flattening and dense layer for output
model.add(layers.GlobalMaxPool2D()) #this is how you flatten if you have variable-sized inputs
model.add(layers.Dense(2, activation='softmax'))
model.add(layers.Flatten())

#View a model summary
model.summary()

#Compile and train the model
#opt = SGD(lr=0.1)
opt = 'adam'
model.compile(optimizer=opt,
              loss=tf.keras.losses.SparseCategoricalCrossentropy(from_logits=True),
              metrics=['accuracy'])

#Fit the model
history = model.fit(train_data, train_labels, batch_size = 1, epochs=15, verbose=1,
validation_data=(test_data, test_labels))

#Save the accuracy on the test data
# convert the history.history dict to a pandas DataFrame:
hist_df = pd.DataFrame(history.history)

#save to csv:
os.chdir('..')
hist_csv_file = 'history.csv'
with open(hist_csv_file, mode='w') as f:
    hist_df.to_csv(f)
```

```
#Save the model so we can make predictions on validation data later
model.save('model1.h5')
```

Supplementary Code S3. ImageJ macro.

ImageJ macro for LDA:

```
run("HSB Stack");
//setTool("oval");
makeOval(1209, 357, 1602, 1578);
makeOval(873, 357, 1938, 1578);
makeOval(873, 357, 1869, 1578);
makeOval(891, 357, 1851, 1578);
makeOval(891, 183, 1851, 1752);
makeOval(891, 183, 1851, 1851);
```

ImageJ macro for CNN:

```
// User selects the working directory
input = getDirectory("Choose Source Directory ");
list = getFileList(input);

// Loop through each file in the directory
for (i = 0; i < list.length; i++) {
    Wholecolor(input, list[i]);
}

function Wholecolor(input, filename) {
    // Open the image
    open(input + filename);

    // Crop the image
    makeOval(550, 350, 1850, 1850);
    setBackgroundColor(0, 0, 0);
    run("Clear Outside");

    // Get average intensity of the whole image
    run("Measure");

    // Save results to a .csv file titled "Whole_Color_Measurements.csv"
    dir = getDirectory("image");
    name = "Whole_Color_Measurements";
    index = lastIndexOf(name, "\\");
    if (index != -1) name = substring(name, 0, index);
    name = name + ".csv";
    saveAs("Results", dir + name);

    // Close the image
    close();
}

run("Clear Results");
```
